# Process evaluation of the school-based Girls Active programme

**DOI:** 10.1186/s12889-019-7493-7

**Published:** 2019-08-29

**Authors:** Trish Gorely, Deirdre M. Harrington, Danielle H. Bodicoat, Melanie J. Davies, Kamlesh Khunti, Lauren B. Sherar, Rhiannon Tudor-Edwards, Thomas Yates, Charlotte L. Edwardson

**Affiliations:** 1 School of Health, Social Care and Life Sciences, Inverness, IV2 3JH UK; 2 University of Leicester, Diabetes Research Centre, Leicester General Hospital, Leicester, LE5 4PW UK; 3 NIHR Leicester Biomedical Research Centre, Leicester, Leicester General Hospital, Leicester, LE5 4PW UK; 4 Leicester Diabetes Centre, University Hospitals of Leicester, Leicester General Hospital, Leicester, LE5 4PW UK; 5 Collaboration for Leadership in Applied Health Research and Care East Midlands, Leicester General Hospital, Leicester, LE5 4PW UK; 6 School of Sport, Exercise and Health Sciences, Loughborough University, Loughborough, LE11 3TU UK; 7 National Centre for Sport and Exercise Science, Loughborough University, Loughborough, LE11 3TU UK; 8 Centre for Health Economics and Medicines Evaluation, Bangor University, Bangor, LL57 2PZ UK

**Keywords:** School-based intervention, Process evaluation, Adolescent female, Physical activity

## Abstract

**Background:**

Girls Active is a physical activity programme, delivered in UK secondary schools, with the aim of increasing moderate-to-vigorous physical activity (MVPA) in girls aged 11–14 years. This study presents the process evaluation as part of a 14-month cluster randomised controlled trial designed to evaluate the effectiveness of the Girls Active programme and which showed no difference in the primary outcome (MVPA at 14 months) between intervention and control arms.

**Methods:**

Quantitative and qualitative data were collected from intervention schools over the course of the 14 month trial. Feedback forms and attendance records were completed at the end of all teacher and peer leader training and review days. At 7- and 14-months, semi-structured interviews were conducted with the lead Girls Active teacher in all intervention schools (*n* = 10) and staff from the intervention provider (*n* = 4) and hub school (*n* = 1). At 14 months, separate focus groups with peer leaders (*n* = 8 schools), girls who participated in the evaluation component of the trial (*n* = 8 schools), and a sample of boys (*n* = 6 schools) were conducted. All participants in the intervention schools were asked to complete an exit survey at 14 months. Teachers (intervention and control) completed a school environment questionnaire at baseline, 7- and 14-months.

**Results:**

The Girls Active programme, i.e., the training and resources, appeared to be well received by teachers and pupils. Factors that may have contributed to the lack of effectiveness include: some initial uncertainty by teachers as to what to do following the initial training, a predominant focus on support activities (e.g., gathering opinions) rather than actual physical activity provision, and school-level constraints that impeded implementation.

**Conclusions:**

Girls Active and what it was trying to achieve was valued by schools. The programme could be improved by providing greater guidance to teachers throughout, the setting of timelines, and providing formal training to peer leaders.

**Trial registration:**

ISRCTN, ISRCTN10688342. Registered 12 January 2015.

**Electronic supplementary material:**

The online version of this article (10.1186/s12889-019-7493-7) contains supplementary material, which is available to authorized users.

## Background

It is well established that participating in regular physical activity (PA) during childhood and adolescence is important for health and well-being [1, 2]. However, data from the UK show that a large proportion of young people are insufficiently active [3], a trend reflected in many parts of the world [4]. PA levels are lower in girls than boys, and there is an observed decline in PA levels during the transition from primary to secondary school [5]. There is a need for effective programmes that promote sustained participation in PA, particularly among girls. Alongside demonstrating effectiveness, there is a need to understand why a programme is effective or not [6].

We conducted a cluster randomised controlled trial to evaluate the effectiveness of the Girls Active programme. ‘Girls Active’ is a physical activity programme, developed by the Youth Sport Trust (YST; a national charity in the UK that works to ensure every child enjoys the benefits of play and sport). The primary objective of the trial was to determine the effectiveness of the Girls Active programme in terms of time spent in objectively assessed moderate-to-vigorous physical activity (MVPA; GENEActiv accelerometer) at 14 months among girls in school years 7–9 (11–14 years old). There was no statistical evidence that Girls Active was effective in changing MVPA at 14 months [7] in the intervention group compared to the control but there was a significant, but small, difference, in favour of the intervention group, at 7 months. Few differences in secondary outcomes were found, but these were sporadic and showed no clear pattern in favour of either group.

Process evaluation is important as it provides information on how or why behaviour changed or did not change, and assists in understanding the outcomes of interventions by exploring how well the intervention was implemented, the experiences of those involved in the delivery or in receipt of the programme, and the effect of the context in which the intervention was delivered [8]. This paper reports on the process evaluation for the Girls Active programme and identifies issues that may have influenced the observed outcomes, and consequently informs both the Girls Active programme going forward and others planning and implementing similar school-based programmes. Specifically, this process evaluation explored what was implemented in each school, enablers and barriers to implementation, and participant (teacher and pupil) perceptions of the programme, including satisfaction, acceptability and enjoyment.

## Methods

Ethical approval for the process evaluation was included within the wider research study approval obtained from the University of Leicester’s College of Medicine and Biological Sciences Research Ethics representative.

### Trial design

The study protocol [9] and a report of the outcomes [7] have been published, along with a final full report to the funder [10]. Briefly, the trial was a two-armed, cluster randomised control trial with 20 schools (10 intervention, 10 control). All girls aged 11–14 years were eligible to be invited to take part in the programme evaluation. Only girls that did not return a parental opt out consent form were eligible to be randomly chosen as one of the 90 girls to be involved in the evaluation. The girls chosen for the evaluation provided verbal assent. Data were collected at baseline, 7 months and 14 months. The primary outcome was objectively measured MVPA (measured by 7 day wrist-worn GENEActiv accelerometer) at 14 months. Secondary outcomes included mean acceleration (overall PA level), light PA, sedentary time, body composition, and psychosocial outcomes at 7 and 14 months.

### The Girls Active programme

The intervention is described in the published protocol [9] and final report to the funder [10]. In summary, the Girls Active programme provides a support framework for schools to review their physical education (PE), sport and PA culture and practices with respect to 11–14 year old girls. It is a flexible programme that schools can mold to match the needs and preferences of their pupils. Although flexible in implementation, there were several key components considered essential to the Girls Active delivery process. School leads for Girls Active attended a one-day training event that introduced the Girls Active programme and associated resources. This day also provided an opportunity for teachers to discuss potential challenges and share ideas. At the training event teachers received a package of supporting resources including action planning guides, case studies, images, video links, and marketing plans aimed at the teachers and the peer leadership and marketing group. All intervention schools completed a self-evaluation and mission analysis to help them review their existing culture and practice for girls’ PA and set out an action plan tailored to their girls’ needs. Following the initial training event, school leads were then encouraged to form a peer leadership and marketing group from Key Stage 3^1^ girls who were seen as leaders, but not necessarily sports leaders or sporty pupils. The role of the peer leadership and marketing group was to influence decision-making in their school, develop as role models, promote PA to other girls, and run peer-led PA sessions and events. Fundamental to the principles of Girls Active is the student ‘voice’ and schools were encouraged to consider the ‘voice’ of the pupil when making important decisions about PA, PE and sport in the school. Schools were challenged to consider a ‘different type’ of student voice that probably would not traditionally have been involved in this type of provision. All intervention arm schools were offered on-going support and mentorship in the form of phone and email support and one-to-one visits, from the hub school (which in this study was the local health and wellbeing school), and the YST. This support provided opportunities to seek advice and to discuss ideas and solutions to overcoming any barriers and challenges that could occur as a result of implementation. Teachers also had access to an online Dropbox folder to share ideas and resources. Approximately halfway through the intervention period, intervention schools were invited to a half-day peer review event to review progress, identify learning, discuss barriers and successes, share ideas and to receive peer-to-peer coaching. This day was organised by the YST and run by the hub school and YST development coach. Following this review event, the schools were required to revise their mission analysis as necessary and submit it to the YST. A further meeting bringing the peer leaders together was held a few months later. This was a celebration event and an opportunity for the peer leaders to meet each other and share ideas between schools. Schools were provided with a capacity payment of £1000 in two £500 instalments. These instalments were released on submission of their mission analysis action plan documentation to the YST. The schools could choose what to spend the money on.

### Data sources

The process evaluation herein relates to the intervention schools only and involved collecting data and information from a variety of sources over the course of the trial. The methods, timings, who the data were collected from, and numbers involved are summarised in Table 1. More detail on these data sources is provided in the following sections and full details can be found in the report to the funder [10]. All interviews and focus groups were conducted by three members of the research team (DH, KH, CLE). Data were anonymised and any comments or observations that could allow individuals of schools to be recognised were removed.

**Table 1:** Process evaluation methods and number of participants involved (adapted from [10])

Type of data	Collected from	Timing	No. of Participants
School details including school environment	Lead teacher, school records, Department of Education school census	Baseline, 7 and 14 months	–
Training event resources	Documents used at the training i.e. timetable, presentation, handouts	During the training event	–
Training attendance and evaluation forms	Training deliverer	At the end of the training	1 training deliverer
	Lead teachers at training	At the end of the initial training day	7 teachers
	Lead teachers at training	At the end of the peer review event	7 teachers
	Peer leaders	At the end of the peer leader event	56 girls
Interviews	Lead teachers	7 and 14 months	10 intervention 8 control at both time-points
	Youth Sport Trust staff members	7 and 14 months	3 staff at each time-point
	The Hub and development coach	7 and 14 months	1 staff member from Hub 1 development coach at both time-points
Focus groups (intervention schools only)	Peer leaders	14 months	46 pupils from 8 schools (range 4–9)
	Subgroups of evaluation sample	14 months	58 pupils from 8 schools (range 4–10)
	A sample of boys	14 months	38 pupils from 6 schools (range 2–8)
Exit survey	Girls of original sample in all intervention schools	14 month	722 pupils

#### Feedback forms

Participants completed evaluation forms at the end of the initial training day, peer review day, and peer leaders event. These forms asked participants opinions on content, perceived learning, delivery, and venue. Attendance at each of the days was recorded.

#### Girls Active Lead teacher interviews

These interviews were semi-structured and guided by an interview schedule devised by the research team. The purpose of these interviews was to explore the implementation of Girls Active in their school, discuss school level changes or events that might have effected delivery of Girls Active, and to explore obstacles and enablers to implementing Girls Active. Further feedback was also sought about the training days, Girls Active resources and programme support. The interviews were conducted at the school at a time convenient for the teacher, and lasted between 17 and 34 min (7 months) and 17–58 min (14 months). Teachers were given a participant information sheet ahead of the interview and signed an informed consent form.

#### Programme delivery and support staff interviews

These interviews were semi-structured and guided by an interview schedule devised by the research team. The purpose of these interviews was to explore what the YST delivered to teachers and how the hub school and YST development coach supported schools. The interviews also explored the obstacles and enablers faced by the Girls Active delivery and support staff. The three main YST staff members who worked on this specific programme were approached via email and interviewed along with the two hub school staff. The interviews with YST staff took place at the YST and the hub school staff were interviewed at their school. All interviews were conducted at a time convenient with the interviewee, and lasted between 49 and 97 min (7 months) and 78 min (14 months – all staff interviewed together). A participant information sheet was provided ahead of the interviews and all staff signed a consent form.

#### Pupil focus groups

Three focus groups with pupils were conducted per intervention arm school at 14 months: (1) peer leaders (*n* = 8/10 schools); (2) a subgroup of 5–8 girls from the evaluation cohort (*n* = 8/10 schools); and (3) a group of 5–8 boys from across years 7–9 (11–14 years old) (*n* = 6/10 schools). Although Girls Active was not aimed at boys, their awareness and views of the programme were sought to explore whether there had been any impacts, positive or negative, upon them. For the sub-group of girls and the group of boys, teachers were asked to choose a variety of pupils from inactive to active but ones who may be more willing to speak up on behalf of their class. Each focus group was guided by semi-structured question schedule devised by the research team and used a combination of individual and group activities and open questions to facilitate discussions around Girls Active and its aims, what had gone on in their school, and how this had impacted upon them. The peer leader focus group also explored any barriers, facilitators, challenges, and opportunities from the peer leader perspective. As the peer leaders may not necessarily have been one of the 90 girls included in the trial, peer leaders were given an information pack to take home to their parents containing a parent/guardian information letter, a participant information letter and an opt out consent form. A similar process was followed for the boys. For the sub-group of girls, consent had already been obtained as part of the main research programme. Written assent was obtained from all pupils before each focus group began. All pupil focus groups were conducted in a quiet space at school during the school day.

#### Exit survey

All girls who were part of the evaluation in the intervention schools were asked to complete an exit survey at the end of the 14 month measurement visit. This survey asked what they understood by Girls Active, whether they had taken part in any Girls Active activities, to list their most and least favourite part of Girls Active, and their opinions on a number of statements about Girls Active.

#### School environment

The lead teachers for Girls Active within each school completed a paper-based school environment questionnaire at baseline, 7- and 14- month follow-up. This questionnaire asked about relevant policies, delivery of different types of sports and PA sessions both within and outside of the curriculum, and school equipment and facilities.

### Analyses

Responses on feedback forms, exit surveys, and the school environment questionnaire were entered into Excel spreadsheets. Descriptive summary statistics were used to explore quantitative responses. Any open-ended responses were entered into the spreadsheet and subsequently grouped into themes.

All interviews and focus groups were recorded, transcribed verbatim, and then analysed using the framework analysis approach. The analysis was carried out by two researchers (TG and CLE). Each school was considered a case. Following familiarisation with the transcripts through repeated reading initial codes were developed to summarise the data within each case. The key questions from the interview/focus group guide provided a starting point for the development of these codes (e.g, intervention components, what Girls Active looked like in each school, activities undertaken, barriers/challenges encountered, benefits of the Girls Active programme), however, we also allowed more inductive coding to capture other data that were relevant to the research aims. Initially the interviews and focus group from one case were coded independently by the two researchers. These initial codes were discussed and agreed before moving on to the remaining cases. Codes continued to be discussed in regular meetings and iteratively refined. As the coding structure was refined, the coding applied to earlier cases was revisited. The final coding structure is summarised in Table 2. Once each case had been coded similarities and differences between cases were explored.

**Table 2:** The final coding structure

Main code – level 1	Sub code – level 2	Sub code – level 3
Training events	Initial training event	Idea provoking
		Sharing experiences
		Lack of implementation timeline
		Too flexible
	Peer review event	Disappointment at cancellation of first event
		Sharing experiences
	Peer leader event	Enjoyable
		More time to dedicate to sharing experiences
Ongoing support	YST support	Valuable
		Non-judgemental
		Additional ideas
		Problem solving
		Mentoring came too late in implementation
	Resource folder	Ideas for implementation
		Used at start
	Action plan	Time consuming
		Confusing
	Money	Funding tight for schools
		Want money to last as long as possible
Activities as part of GA	Physical activities	Girls only clubs
		New clubs (short duration)
		Taster sessions
		Activity days/one-off events
	Support activities (not PA)	Loyalty card and prizes (reward schemes)
		Suggestion box
		Changing physical environment
		Clothing for peer leaders
		Posters
		Bulletin board
		Surveys/canvassing opinions
		Marketing through assembly
	Peer leader group	Chose sporty/active/engaged girls
		Teacher reluctant to delegate
	Meetings	Peer leader group
		Form groups
		Peer leaders with head teacher
	PE related	Looked at content
		Uniform review
Barriers/challenges	Logistics	Timetable restrictions
		School buses
		Attendance at training days
		Cost
		Access to gym/space
		Providing variety
		Creating widespread awareness
		Getting peer leaders together
	Lack of time	Life gets in the way
		Takes a lot of time
		“competing demands” (wider school commitments)
		Wish had dedicated more time for programme
	Curriculum	Fit of changes with national curriculum
	Interpersonal	Peer leaders feel judged
		Peer leaders feel responsible if can’t deliver what other want
		Peer leaders feel pressure to meet diverse needs
		Girls feel judged
		Fear of losing friends
	Attitudes	Gender stereotypes
		Getting girls to come
		Self-conscious
	Buy-in from other teachers	Not viewed as priority
	Organisation	Knowing what to do
		Lack of timeframe for implementation
		Having other teams to play
		Cancellations
		Equipment
Facilitators	Support	From teachers (Peer leaders)
		Other teachers and senior management (teachers)
		External (from YST)
	Feeling part of something bigger	
	Being monitored	
What is Girls Active?	Choice	
	Promoting girls activity	Speaking out for girls
		Encourage girls to do sport
		Showing girls can do it
		Motivating girls
		Getting opinions
	Measurement days	Watches (GENEActiv accelerometers)
		Find out how active girls are
	Activities	Loyalty cards
		Prizes
		Fun activities
	Sexist	Not all boys play sport – should be given this opportunity too
	Better kit	
	Changing girls	Get girls fitter
		Get girls more active
		Build confidence
		Change PE
Impact/benefits	Peer leaders	Seeing changes
		Role model
		Sense of responsibility
		Friendships
		Get out of class
		Confidence
		Learning from other schools
		Leadership
		Help others
		Learning new PA skills
		Developing organisation and planning skills
	PE	Less moans
		Talking about health
		Choice
	None yet	Late started
		Needs time to see them
	Enjoyment	
	Increase attendance at clubs	New faces at clubs
	Good opportunity	
	Boys	Interest from boys

Findings from this process evaluation are presented in a mixed methods format using the quantitative and qualitative data to describe what was implemented in each school, enablers and barriers to implementation, and participant (teacher and pupil) perceptions of the programme, including satisfaction, acceptability and enjoyment. These are addressed under three main headings in the results section: engagement with, reach and dose of the intervention; opinions and experiences of the intervention; and, impact of the intervention.

## Results

### Engagement with, reach, and dose of the intervention

#### Training events

Eight out of 10 schools attended the initial training event (April 2015), although two schools did attend a shorter ‘mop up’ event, and seven out of 10 schools attended the peer review and peer leader (February 2016) events. The YST reported that the events were delivered largely as planned, although the peer review event for teachers took place 2 months later (December 2015) than the original date (September 2015) due to only a few teachers being available for the original date.

#### Ongoing support (resource folder, action plans, Dropbox, YST visits, hub school, funding)

The teachers reported using the resource folder at the start of the programme to generate ideas for implementation however, use was not sustained into the programme. For some teachers, use may have dropped off as teachers and peer leaders became familiar with the materials, and perceived less need for the resources as they started to have their own ideas of where to take the programme within their school.

*“We used a lot at the start, not really at all towards the end”* (Teacher; School 2)

*“It’s probably not been something we’ve really referred a lot to as we’ve gone along but for a starting point it’s definitely something useful”* (Teacher; School 3)

*“At the very start of the project, when you looking at the very first action plan and looking at kind of cases and things...you know, it kind of...okay, what can we do, but other than that, it’s not been used at all … .[I: is there a reason for that?] No … I’ve just got on with what I thought that we should do here and made if school specific to us and generally just not really looked in it”* (Teacher; School 6).

All schools submitted both their action plans (Table 3). However, four schools did not submit their first action plan, until after the 7 month follow-up evaluation visit (8 months after the initial training). This meant they did not receive their first funding instalment until after the 7 month follow-up evaluation visit. Four schools did not submit their second action plan until the time of the 14 month evaluation visit. Six schools received the first funding by the end of the summer term 2015 (July), 3 months following the initial training day. The second funding instalment was received in January 2016 by six schools and four schools did not receive their instalment until May 2016, around the time of the 14 month evaluation visit. It is evident from these dates that funding in some schools was received late on in the intervention cycle and therefore the schools had limited time/opportunity to spend it as part of the Girls Active programme. Many of the schools did not spend any, or only a very small amount of the funding, during the 14 month programme.

**Table 3:** Summary of when each intervention school submitted their mission analyses and received funding (adapted from [10])

School	Mission analysis 1	Funding 1	Mission analysis 2	Funding 2
School 1	December 2015	December 2015	April 2016	May 2016
School 2	May 2015	July 2015	December 2015	January 2016
School 3	November 2015	May 2016	April 2016	May 2016
School 4	December 2015	December 2015	December 2015	January 2016
School 5	December 2015	January 2016	December 2015	January 2016
School 6	June 2015	July 2015	January 2016	May 2016
School 7	June 2015	July 2015	December 2015	January 2016
School 8	June 2015	July 2015	April 2016	May 2016
School 9	June 2015	July 2015	January 2016	January 2016
School 10	June 2015	July 2015	December 2015	January 2016

To facilitate the sharing of ideas and resources teachers had access to a Dropbox folder. It appeared though that this was underutilised with teachers forgetting about it or reporting that it was not updated regularly.

“I have looked in there a couple of times but no one seems to have put anything in” (Teacher; School 8)

“I know there’s the Dropbox, again, it’s trying to get the time to even think about getting into the Dropbox to look” (Teacher; School 6)

Not all schools accessed the phone call support offered by the Hub school. However, all schools had at least one visit, sometimes two, from the YST development coach, although these did not take place until eight - 10 months into the intervention.

#### Implementation in the school

One of the first tasks teachers were encouraged to do as part of the intervention was to establish a girls peer leadership and marketing group. Eight out of 10 schools achieved this, although the timing for establishment of the groups varied (e.g., six schools established the group by the 7 month evaluation visit, whereas in two others the group had only recently been established by the time of the 14 month evaluation visit). One school did not formally set up a group but did have a group of girls that would help out with certain tasks when needed. However, teachers often reported a reluctance or difficulty with delegating tasks to them. When selecting girls for the peer leadership and marketing group, the teachers were encouraged to select girls who were perceived as leaders and therefore in a position to positively influence their peers, and to not necessarily choose girls who were already involved in sport and physical activity. Although many of the teachers reported trying to comply with this type of selection, a lot of the chosen peer leaders reported that they were sporty or had an interest in physical activity and sports.

Table 4 summarises the implementation activities within each school; information which was gathered through interviews and focus groups. All but one school conducted some support and/or physical activities during the 14 month programme. Many of the activities implemented in the schools were ‘support’ activities, including questionnaires to canvass opinions from girls regarding current PE, sport and PA provision (*n* = 5 schools), changes to PE kit policy (*n* = 3 schools), reward schemes (*n* = 5 schools), marketing and promotion of Girls Active through assemblies (*n* = 5 schools), posters (*n* = 5 schools), notice boards (*n* = 3 schools), and purchasing branded hoodies for the peer leaders (*n* = 2 schools). Some schools ran taster activity sessions where pupils could come along and try different activities (*n* = 2 schools) and/or new sports/activity clubs (*n* = 4 schools), but these were usually only for a limited time (e.g., a few weeks). Two schools delivered one-off events, for example sports relief fund raising events to coincide with national campaigns.

**Table 4:** A summary of the activities implemented within each school at 7 and 14 months

	Support Activities	Physical activities
School 1	*By 7 months:* presentation to staff to try and find other members of staff to get involved. *By 14 months:* peer leaders had held meetings, organised a suggestion box, hoodies, looked at PE content, talked to form groups.	*By 7 months:* none. *By 14 months:* None but girls only extra-curricular club and a loyalty card for attendance planned.
School 2	*By 7 months:* teacher recruited another member of staff to assist, peer leader meetings once/week, posters advertising clubs, survey of opinions, looked at after school transport issue, looked at painting changing rooms, peer leaders went to head teacher to explain what they were planning. *By 14 months:* inspirational posters for changing rooms and every tutor room, PE teacher promotes Girls Active in PE lessons, weekly bulletins.	*By 7 months:* new clubs (cheerleading, Zumba, dance, basketball) with external coaches delivering these (no longer operating at 14 months). *By 14 months:* just started a “Healthy Life Club” (once a week, walk/jog/run/talk to music) and an activity loyalty card.
School 3	*By 7 months:* peers leaders launch Girls Active in assembly, questionnaire to girls, girls ‘drop in’ sessions, designed new netball kit. *By 14 months:* reviewed PE kit policy and petitioned to get changes to the kit but head teacher did not approve changes, meeting between peer leaders and senior leadership team (planned), hoodies for peer leaders (planned next term)	*By 7 months:* nothing. *By 14 months:* one-off sport relief event (ninja warrior course in the gym - 200 attendees), non-uniform sports day, yoga, dance and boxercise sessions (with lights turned off so no-one can see them doing it), changing some activities to make them girl friendly (planned next term).
School 4	*By 7 months:* asked girls that weren’t involved in sport what they wanted to do (teacher went round as many classes as possible with pen and paper), feedback from school stakeholder group (boys and girls); ensuring clubs take place indoors (in response to feedback); *At 14 months:* teacher plans to set up a reward scheme for regular attendance at clubs, plans for the Girls Council to present to Senior Leadership Team.	*By 7 months:* girls only after school fit club. *At 14 months:* after school rounders club and dance club.
School 5	*By 7 months:* marketing during form time, assemblies, video displays around school, stand up banners with reminders to be active and where they can access information, designed a club board for the gym using local information on clubs. *By 14 months:* Nothing else done.	*By 7 months:* taster and satellite sessions after school. *By 14 months:* one-off Sports Relief fitness challenge and peer leaders were planning a Zumba party.
School 6	*By 7 months:* nothing. *By 14 months:* assemblies, meetings, asking people what they wanted, posters/bulletins.	*By 7 months:* nothing. *By 14 months:* “Thursday Club” (after school, different activities could be tried, 15–30 attend), peer leaders PE lesson.
School 7	*By 7 months:* external person did a session on marketing, meetings, planning of loyalty scheme. *By 14 months:* surveys of other pupils, bulletin board, posters, meetings, assembly, introduced loyalty scheme.	*By 7 months:* already had lots of clubs so decided not to introduce more. *By 14 months:* one-off taster sessions (~ 25 girls attend) e.g., Zumba, hula, yoga (spread throughout one term), about to run a “Girls Active day” (June 2016) for all girls in the school with lots of tasters sessions and quizzes and prizes e.g., cheerleading, trampolining, Zumba, boxercise.
School 8	*By 7 months:* questionnaire to all girls, changes to PE kit policy, whole school reward initiative. *By 14 months:* a second whole school reward initiative was being implemented from Easter where students could receive a range of different rewards for participation in any clubs (not just physical ones) e.g., free skipping ropes, footballs, basketballs, cricket stumps, art packs, lunch queue jump.	*By 7 and 14 months:* no additional physical activities (i.e., clubs) delivered.
School 9	*By 7 months:* peer leader hoodies, assembly to girls, changes to PE kit, meetings, teacher spoke to staff at a CPD event, questionnaire to girls, external badminton player came in and did inspirational talk to 20 girls from years 8 and 9 (those who were not engaged but had influence), weekly challenge to say something positive to someone in their lesson, using staff as role models e.g., if they have done something active display it on the school TV screens. *By 14 months:* posters and MOODLE pupil online bulletin boards.	*By 7 months:* dodgeball and gymnastics club (around 30 attendees at each), fitness challenge in form time e.g., plank and wall sit challenge (whole school). *By 14 months:* reward card scheme, activity day, promotion of existing clubs.
School 10	*By 7 and 14 months:* nothing done.	*By 14 months:* nothing done.

The school environment questionnaire revealed that only two schools had an increase in girls only sports/activity clubs. No other changes were reported in sports/activity clubs offered and access to facilities and equipment. Some policy changes were noted but these were also identified through the teacher interviews.

The exit survey revealed that Girls Active implementation did not have the intended widespread reach within the schools after 14 months. For example, 36% of girls reported participating in ‘Girls Active’ activities and although 79% of girls had ‘heard of Girls Active’ they mainly associated this with the evaluation activities (i.e. study questionnaires and wearing the accelerometer). Furthermore, only 25–48% felt that their opinions and ideas had been sought and 40% felt that the teachers and peer leaders had encouraged them to take part in Girls Active activities.

Overall, implementation was slow, often towards the end of the evaluation period and was not to the extent outlined in action plans. Teachers themselves were aware that they had not achieved all that they set out to do.

*I don’t feel like I’ve done it well enough … in hindsight I should have been more proactive in making sure I made the time, too often I thought I need to do that and I’ve not done it. And I know deep down that it will be good and it will work … I don’t think I’ve made as much use of it as I could have done.* (Teacher; School 1)

### Opinions and experiences of the intervention

#### Training events

Feedback on all the three training events was very positive (see Additional files 1,2,3,4, and 5), with attendees reporting that they were well organised, informative, great for sharing experiences, and idea provoking.

*“That training day* [initial training event]*, in my opinion, was really inspiring. I found it really brilliant. I came back really buzzing, can't wait to get things started … … I thought I got loads out of it, this is how we can do this, this is how we can do that, came back and had a really positive meeting with the department”* (Teacher; School 8)

*“*[the peer review event was] *Useful to share and hear what other people were doing but also quite nice for sharing what we've done as well and other people being interested in it … really good sharing opportunity and appreciated to know that either you are ahead of what other people are doing, or you have helped somebody to be able to get where they are or there are certain things that you are finding difficult”* (Teacher; School 7)

*“they loved the activities and things* [at peer leader event]*, it was good team bonding stuff”* (Teacher; School 9)

Although the training events were well received, the teachers highlighted some potential issues that could have impacted on implementation of the programme in their own schools. Firstly, the initial training event did not necessarily leave teachers with a clear plan or timeline for next steps for implementation. This was reflected in the quantitative evaluation of the training with 71% of teachers only feeling ‘somewhat confident’ to be able to go back to their school and implement Girls Active.

*“I didn’t feel, probably when I came back home, and they asked me the next day, oh, so what do we have to do? I kind of felt a bit like, I’m not really sure. I knew why we were doing it, but then I was a bit like, so what do we actually do?”* (Teacher; School 9)

Secondly, the date for the peer review event had to be changed due to low attendance numbers and this took place two months later than planned. Several teachers expressed their disappointment at this, particularly as they were hoping it would highlight the next steps for implementation.

*“It's a shame that that was cancelled because that’s what I was using for the next step”* (Teacher; School 8)

Finally, whilst the peer leader event was enjoyable for the leaders and 98% rated the event as ‘very good’ or ‘good’, the teachers felt that more time should have been dedicated to the girls from the different schools sharing ideas and formulating plans for implementation.

*“I just think the girls themselves they could have spent a bit more time finding out from each other what they did. They got a lot from it but I don't think they actually got to share their ideas much with each other which I think would have been useful because then for my girls they maybe could have picked up ideas and picked up enthusiasm from what the other girls had done and thought oh that will be a really good idea we could go and do that”* (Teacher; School 1)

#### Ongoing support (resource folder, action plans, Dropbox, YST visits, hub school, funding)

Many of the teachers mentioned that the resource folder was very helpful for generating ideas for the types of things they could implement within their school. Several reported that they had taken ideas from the case studies and used these to help the peer leaders to understand the sorts of things they could do and to encourage the girls to work independently.

*“So that actually stimulated a lot of conversation about our uniform and kit. There was a lot of examples in there about that”* (Teacher; School 3)

*“I thought the packs were useful, because there were a load of case studies in there, from other schools. So there was a Race 4 Life case study, that we took, that we’re doing, so we’ve applied to be a Race 4 Life school”* (Teacher; School 5)

*“I think that enabled the girls that had taken part as the girls group to actually do everything themselves so therefore I've not...they've come to me and said, shall we do this?”* (Teacher; School 7)

Many of the teachers reported that the first action plan was time consuming to complete and there was a lack of understanding of the sections on the form. The second action plan form was modified in response to the teacher feedback and the teachers found it easier to complete.

*“Oh it's taken ages to complete and sometimes we look at it and we go does that really mean that? Yeah it's taken a lot of time to go through”* (Teacher; School 2)

*“I didn’t understand really, what they were asking. So like, so kind of strategies into action, does your plan drive everything you do? What plan? What kind of things do you mean by that?”* (Teacher; School 9)

*“The action plan that they gave us the second time around was a lot easier. A lot easier … ”* (Teacher; School 6)

Although the teachers spent very little money, they did feel that this aspect was important and were very appreciative of the funding. Teachers felt that because funding within schools for sports and physical activities was tight, they wanted to plan appropriately on how to spend it and to make it stretch across several school terms.

*“If we can make that money last for a long period of time, then that is, you know, if we are only using a real small portion of it per year, we would make it last maybe two or three years”* (Teacher; School 7)

*“we’ve been very lucky that we’ve got this money through the Girls Active, but we want it to last as long as possible. Once that money has gone, it’s not then sustainable, so we’ve only had one coach in. We’ve only spent a little bit of money, but it means that in September we can get another coach in, so it’s like spreading it out and we can sustain this now for a couple of years”* (Teacher; School 6)

The teachers felt that the support visits conducted by the hub school and the YST development coach were extremely valuable and non-judgemental. They also encouraged them to progress further with implementation of the intervention; for example, through the generation of additional ideas, reinforcement, problem solving, and the thought of having someone monitoring them.

*“She’s been so useful for bouncing ideas off and, kind of, yes, just giving feedback on how perhaps I could have approached something that would have helped me out. It’s just been really useful because she is so casual about it so I haven’t felt judged as well. If I’ve said, oh, no, this hasn’t gone very well or anything, you know, she’s been really good and just that general having someone to feedback to, ask questions to, it’s been really useful”* (Teacher; School 5)

*“She’s someone who you know is monitoring you, without you feeling like you’re being monitored. I feel like she’s supporting, rather than pointing the finger at things that you may not have done. Without her, I’d be left to my own devices, and without being reminded and in contact with these people, I don’t know where that would feature on my to do list”* (Teacher; School 8)

However, several teachers highlighted this type of support was needed earlier on in the programme to facilitate the development of ideas and timelines for implementation early on.

*“You almost need that support throughout the project to keep you going and to keep the motivation and the self-belief...yeah, you are doing the right thing and I think without that, I don’t think it would work.”* (Teacher; School 6)

#### Implementation in the school

Overall, Girls Active was viewed positively and lead teachers, peer leaders and girls reported enjoying being involved.

*“We thought it was a brilliant project that our girls would be on-board with this and it was a good thing for the school to be honest”* (Teacher; School 2)

*“Honestly I think Girls Active project is such a good one. I think because anything we can do to get girls involved in PE is so important and I think it’s just the way to do it like getting the girls to do it themselves so that it spreads the word that way; really good”* (Teacher; School 5).

*“It's such a good opportunity that I hope other schools realise what they're having and what they've got”* (Teacher; School 7)

Some teachers reported that they had involved their senior leadership team and that implementation was made easier when the senior leadership team was supportive. Teachers also felt that although, as mentioned previously, the school visits from the YST development coach came too late in the programme, they were extremely useful to progress implementation in terms of clarification on direction, ideas, problem solving and general motivation and support.

*“Just general support really within the department...you know, they’ve all been really supportive of what we’re doing”* (Teacher; School 6)

*“it* [the YST visits] *definitely, kind of, prompted me just to go, actually I could do this easier this way, so it was just someone clearing my mind really. So it was just, kind of, someone calming, really just to re-set me, my goals, what I want to do, cut it down, do the basics, then build on that. And it massively just really helped me re-evaluate”* (Teacher; School 4)

However, they did experience numerous challenges during implementation of support and physical activities. These included finding adequate time to dedicate to planning and delivery, lack of engagement from other staff and the senior leadership team, the programme not being viewed as a priority within the school, wider school level commitments (e.g., OFSTED visits, amalgamating schools and increasing school size), and lack of a timeframe for the implementation of elements of the programme. There were also challenges finding time slots for peer leaders to meet, meaning that meetings were often short, and consequently discussions and planning of activities was spread over many weeks.

*“Just trying to get everything done really, managing your time. It’s an extra project that we took on and it’s just finding those extra bits of time to kind of do it alongside everything else that you’ve on”* (Teacher; School 6)

*“It stalled slightly after that because Ofsted hit in the last week of term in the December so then when we came back in January there was a lot of stuff that we had to implement. I had to put the Girls Active stuff a bit on the back burner”* (Teacher; School 1)

*“I'm not quite sure [our head] appreciates how big it is and how big it can be. I think if she did know, she might be more inclined to give us a bit more time. But, even then, time gets taken off of us like that. She’d say, not today, you can't do that today, you’ve got to do this instead”* (Teacher; School 8)

*“Yes, I haven’t had much backing.. It’s like you’re on your own and you actually haven’t got the support to go anywhere else”* (Teacher; School 5)

*“Just maybe give a bit of a timeframe because that’s the only trouble, you find that it’s not necessarily the priority”* (Teacher; School 6)

*“We [peer leader meetings] meet usually once a week, it's during registration. We have had an hour's meeting as well which … The AM registration time is very short, so to try and ram everything in and to find out what we need to know is really quite short”* (Teacher; School 2)

Although the peer leaders felt that it had been a positive and worthwhile experience to be part of Girls Active, some reported feeling pressure at being able to come up with new ideas and deliver on the changes that were requested by their peers. Others reported that being a peer leader had meant that at times they had not been able to do something else.

*“some of them [girls] like they're desperate to get improvements and they want changes in the school and I think they're relying on us because they know we've been like asking people and like going to the principal and trying to like get these things to happen”* (Peer leader; School 3)

*“I think there’s been some things that you’ve had to sacrifice and things but I don’t think it’s been anything really hard at all … Like clubs and things that you might have normally been going but you thought, actually I’m not going to do that because I’m doing this”* (Peer leader, School 5)

### Impact of the intervention

Although Girls Active was not implemented to the extent planned during the evaluation timeframe, the teachers did feel that what they had already, or were starting to, implement within their schools was making some positive impact in terms of girls’ motivation for being active and attendance levels at clubs.

*“the first time we did swimming was in the first week of this term and there were 18 students. Last week there were 43 … . I've never seen some of the girls in year eight before, now I know who they are because they're regularly coming to clubs … Why have you started to come to swimming club? I want to go to Planet Bounce* [talking about logging activities and the potential for pupils to win a free trip to Planet Bounce]” (Teacher; School 8)

*“And we’ve now got dodgeball on the extra-curriculum, and the amount of people that come to those clubs is fantastic. We’ve got 30, which is just brilliant, and the same with gymnastics. We don’t normally get that much attendance on after-school”* (Teacher; School 9)

Girls Active particularly encourages the engagement of inactive girls and some of the teachers reported that the sports and activity clubs were attracting a small number of girls who previously had not attended.

*“actually I've seen girls, the ones that haven't necessarily come to a club before, we've then seen that they have come to that and gone, oh it's actually all right, and then gone to something different”* (Teacher; School 7)

*“with the likes of the dodgeball and those kind of activities I’ve seen quite a few new faces”* (Teacher; School 9)

*“the ones that came to the club, they are...I can think off hand, probably three or four that don’t come to any club at all”* (Teacher; School 2)

The teachers felt that impact would continue to build as implementation increased into the coming school terms and once the programme influenced the whole school ethos to a greater extent.

Girls Active also appeared to have a positive impact on personal development of the peer leaders. Several peer leaders reported developing skills in organisation and planning, as well as improving their confidence and problem solving skills.

“*If we were in a job that we needed to organise or plan something or make our own ideas it kind of helps because we’ve had practise putting stuff together and like resolving problems that we’ve faced”* (Peer Leader; School 3)

*“It makes you feel good about yourself because you have responsibility, have responsibilities, they can help you feel confident”* (Peer leader; School 5)

Some girls and peer leaders felt that their enjoyment of PE, sports and physical activity had increased as a consequence of teachers allowing them to have an opinion/voice on what is offered and how it is offered.

*“The PE teachers give us a choice of what we want to do in PE rather than telling us what we’ve got to do. It makes us enjoy PE more because it’s doing something that we want to do rather than being… Forced to do it”* (Girls Subgroup, School 4)

In the exit survey 46% of girls reported liking physical activity a bit or a lot more and 45% reported liking sport and PE a bit or a lot more, which reinforced these feelings.

Teachers reported that Girls Active did not affect the boys in any significant way and that the boys were interested in what was going on with Girls Active, mainly around the evaluation and why were the girls getting rewards etc. The focus groups with boys generally reflected this, however a small number of boys questioned why boys were not offered a similar programme given that not all boys are active. One boy felt that Girls Active was sexist.

*“Why do the girls...can I wear one of those? so I have explained to a couple of boys the reasons behind why we are doing the Girls Active and why the driving is towards girls”* (Teacher; School 7)

*“A lot of the boys have made comment that they weren’t allowed to be involved in it. They wanted to be involved in something and why are the clubs only for girls, why is there more girl clubs than boys”* (Teacher, School 4)

*“[What is girls active?] Sexist. Because why don’t boys get the opportunity, because there are some boys that don’t play sport and there are some girls that play sport, so I don’t see why it should be different for girls? Yeah, it’s good to get girls, like, active, but, like I say, it’s the boys as well that need to be active as well, it’s not, like, girls are just going to change the world by doing sport”* (Boys focus group; School 7)

A couple of the teachers did suggest that the increase in girls’ clubs had impacted on boys in a negative way as they were unable to access the facilities while the girls were using them.

*“they’ve lost some of their time that they’ve been out on the pitches”* (Teacher; School 3)

## Discussion

This paper presents the findings of the process evaluation from the evaluation of the Girls Active programme and may explain why Girls Active did not lead to significant changes in objectively measured physical activity over the evaluation timeframe. There was support from teachers and pupils for Girls Active and what it was trying to achieve. The programme was implemented to some extent in all but one of the schools but it was clear that they did not achieve many of the planned activities listed on their action plans. The training and resources provided were positively received and considered informative and useful. Teachers perceived that Girls Active had made a positive impact on girls’ motivation, enjoyment and attendance even within those girls who did not necessarily engage previously, which is positive as Girls Active is particularly aimed at those with low activity levels. Teachers also said that they would continue to implement aspects of the programme following completion of the trial which demonstrates the teachers’ enthusiasm for the programme. The process evaluation identified a number of factors that may have contributed to the non-significant findings in objectively measured physical activity.

The Girls Active programme has a number of core components that are standardised across all schools (e.g., training, resource provision) but some components (e.g., giving pupils a voice, ensuring activities offered are attractive to girls) are based on schools themselves developing their own programme of implementation that suits their particular context, and while this is a strength it also presents challenges. The flexibility created by having choice in activities and timing of delivery on occasion created uncertainty. Without milestones or deadlines teachers found other priorities took over and the programme drifted, potentially explaining why schools did not achieve everything they set themselves in their action plans. Greater support earlier in the intervention implementation phase and the setting of milestone dates early were suggested as ways to counter these challenges. Furthermore, providing teachers with the dates for all training or review days at the beginning of the programme would help lead teachers to plan their cover and potentially act as motivators for progressing implementation so that they had something to report.

The types of activities undertaken in the schools may have contributed to the lack of change in MVPA observed. As noted in the results many of the activities chosen by schools were ‘support’ activities aimed at finding out what girls in the school wanted or targeting cultural change. While these are encouraged as important steps within the Girls Active programme and are important for the long-term sustainability and impact of the programme, the potential for impact on actual PA behaviour is limited in the short term. Relatively few new opportunities for PA were implemented or embedded during the intervention and, when they were, these were often one-off events or only for a limited time (e.g., several weeks) and thus did not result in sustained opportunities for participation. It is not clear whether encouraging schools to give more attention to the provision of actual physical activity earlier in the programme would have resulted in changes in MVPA but it is worthy of consideration going forward.

Within the Girls Active framework, it was expected that the peer leaders would take on substantial responsibility for organisation and planning, including helping to lead new physical activities. This is a large and time consuming task. Combining this with the challenges in bringing the peer leader group together for planning (for example, many peer leader groups were only able to meet once a week or once a fortnight for 15–25 min), and constraints on lead teachers and pupils time, meant that intervention activities, especially physical activities, took many weeks and sometimes months to implement. Furthermore, the peer leaders did not receive any formal training for their role and it was over halfway through the intervention period when they were brought together with the opportunity to share ideas at the peer review day. A more structured approach where peer leaders are provided with session plans that they could choose from may help this inexperienced group maximise the time available. Although, peer-based physical activity interventions have been shown to be successful in increasing physical activity [11], they usually involved a more structured approach with training and a definitive plan of what and how to do things. More recently, a number of peer-based physical activity interventions are being evaluated and it will be interesting to compare the results of these trials with the present trial [12, 13]. Sustained behaviour change likely requires both opportunities for activity and wider school environment changes emphasised as part of Girls Active.

Resources within the intervention did not appear to be fully utilised by schools. The resource folder was considered helpful in the early days of the intervention but then use tapered off. Hub support and Dropbox materials were only accessed occasionally and appeared to have been forgotten about in many cases, and the capacity funding was generally not fully spent within the intervention period. These findings would suggest that teachers need proactive reminders about the support available and perhaps encouragement to spend the capacity funding to launch Girls Active in a concentrated manner. It may also be helpful to send novel material to add to the resource folder throughout the programme. If such material is to be uploaded for access from a centralised “cloud” storage then direct contact to teachers at point of upload is likely required to act as a reminder and encourage access. The visits by the development coach were viewed very positively, and perhaps introducing these earlier in the programme, and scheduling them more frequently over a longer period of time would provide not only the valued support but also provide direct reminders and impetus for continued engagement with resources throughout the Girls Active programme.

### Strengths and limitations

This paper reports on the in-depth, process evaluation of the Girls Active programme. The perspectives of multiple stakeholders (i.e., participants, teachers, programme delivery staff) were canvassed using both qualitative and quantitative methods. There is potential for bias in the selection of participants in the various pupil focus groups, as although teachers were asked to select a variety of both active and inactive pupils to represent a variety of views and experiences, we cannot be sure that this happened. There is also the potential for social desirability bias in all the evaluation components, meaning that participants may have reported what they feel the researcher wants to hear. It was not possible to blind the interviewers to the intervention status of the schools which may have created the potential for interviewer bias.

## Conclusion

Based on this evidence, the failure of the Girls Active programme to elicit significant MVPA behaviour change in the intervention arm at 14 months can probably be attributed, at least in part, to some initial uncertainty in schools as to what to do, a predominant focus on support activities rather than provision of actual physical activity opportunities, and school level constraints (e.g., teacher time, other priorities) that led to time delays in the implementation of intervention components and activities. The lack of behaviour change does not seem to be associated with the training or resources, which appeared to have been well delivered and favourably received. Flexible school based approaches such as Girls Active need to consider more contact with teachers in the early implementation phase and regular ‘monitoring’ throughout. In addition, strategies are needed to support teachers to keep such interventions running in the face of pressures from wider school priorities and changes.

## Additional files

Additional file 1: **Table S1.** Teacher (*n* = 7) ratings for each of the sessions delivered within the initial training event (DOCX 22 kb)

Additional file 2: **Table S2.** Teacher (*n* = 7) ratings for peer review event (1) (DOCX 22 kb)

Additional file 3: **Table S3.** Teacher (*n* = 7) ratings for peer review event (2) (DOCX 22 kb)

Additional file 4: **Table S4.** Ratings for the peer leader day by the girls (n-56) (1) (DOCX 16 kb)

Additional file 5: **Table S5.** Peer leader (*n* = 56) ratings of the Girls Active Leaders Event (DOCX 15 kb)

## Data Availability

Owing to the use of opt out consent, and not including any specific data sharing information in the participant and parent/guardians information sheets, there are no data that can be shared publically. Please contact the corresponding author for further details.

## Abbreviations

MVPA: Moderate to vigorous intensity physical activity
OFSTED: Office for Standards in Education, Children’s Services and Skills
PA: Physical activity
PE: Physical education
UK: United Kingdom
YST: Youth Sport Trust

## Footnotes

1 A Key Stage is a stage of the state education system which in England, Wales and Northern Ireland sets the educational knowledge expected of students at various ages. Key Stage 3 includes children who are 11–14 years of age and usually covers the first 3 years of secondary school.
