# Supplementary material for: Process evaluation of the school-based Girls Active programme
Source: BMC Public Health. 2019 Aug 29;19:1187. doi: 10.1186/s12889-019-7493-7 (PMC6716893; doi:10.1186/s12889-019-7493-7)
Supplement: Supplementary file 1 — Table S1. Teacher (n = 7) ratings for each of the sessions delivered within the initial training event (DOCX 22 kb) [file 12889_2019_7493_MOESM1_ESM.docx]

**Additional File 1.**

**Supplementary Table 1. Teacher (n=7) ratings for each of the sessions delivered within the initial training event**

|  | Poor | Satisfactory | Good | Excellent |
| --- | --- | --- | --- | --- |
| Why PE and Sport for adolescent girls - the challenges and principles | 0% | 0% | 57% | 43% |
| Analysing PE and sport for girls, considering motivations | 0% | 0% | 57% | 43% |
| Marketing PE and sport for girls | 0% | 0% | 57% | 43% |
| Resources and support for the pilot project | 0% | 0% | 57% | 43% |
| Girls Active Self Review and action planning | 0% | 0% | 71% | 29% |
| Girls Active process, evaluation and next steps | 0% | 0% | 57% | 43% |
