# Supplementary material for: Process evaluation of the school-based Girls Active programme
Source: BMC Public Health. 2019 Aug 29;19:1187. doi: 10.1186/s12889-019-7493-7 (PMC6716893; doi:10.1186/s12889-019-7493-7)
Supplement: Supplementary file 4 — Table S4. Ratings for the peer leader day by the girls (n-56) (1) (DOCX 16 kb) [file 12889_2019_7493_MOESM4_ESM.docx]

| **Additional File 4.**  **Supplementary Table 4. Ratings for the peer leader day by the girls (n=56)** | | | | |
| --- | --- | --- | --- | --- |
|  | To a great extent | To some extent | To a little extent | To no extent |
| I have a better understanding of the Girls Active programme... | 45% | 55% | 0% | 0% |
| I have a better understanding of what motivates girls... | 36% | 55% | 7% | 2% |
| I have a better understanding of the benefits... | 64% | 32% | 4% | 0% |
| I have gained new ideas to help me... | 36% | 59% | 5% | 0% |
| I have improved my skills to help me... | 45% | 50% | 5% | 0% |
| I have been inspired and motivated to encourage... | 41% | 52% | 7% | 0% |
| I have gained more confidence to influence... | 48% | 45% | 5% | 2% |
| Do you feel clear on your next steps | 42% | 51% | 7% | 0% |
| Are you in a position to take action | 44% | 53% | 3% | 0% |
| Do you feel supported by your teachers | 80% | 20% | 0% | 0% |
