# Supplementary material for: Process evaluation of the school-based Girls Active programme
Source: BMC Public Health. 2019 Aug 29;19:1187. doi: 10.1186/s12889-019-7493-7 (PMC6716893; doi:10.1186/s12889-019-7493-7)
Supplement: Supplementary file 5 — Table S5. Peer leader (n = 56) ratings of the Girls Active Leaders Event (DOCX 15 kb) [file 12889_2019_7493_MOESM5_ESM.docx]

| **Additional File 5.**  **Supplementary Table 5. Peer Leader (n=56) ratings of the Girls Active Leaders Event** | | | | |
| --- | --- | --- | --- | --- |
|  | Very good | Good | Average | Poor |
| Venue | 67% | 27% | 6% | 0% |
| The instructors | 65% | 33% | 2% | 0% |
| Format of delivery | 48% | 44% | 8% | 0% |
| Pace of delivery | 52% | 38% | 8% | 2% |
| Meeting your needs | 50% | 42% | 8% | 0% |
| Overall, how would you rate your experience of the Girls Active Leaders Event? | 57% | 41% | 2% | 0% |
