# Supplementary material for: Process evaluation of the school-based Girls Active programme
Source: BMC Public Health. 2019 Aug 29;19:1187. doi: 10.1186/s12889-019-7493-7 (PMC6716893; doi:10.1186/s12889-019-7493-7)
Supplement: Supplementary file 2 — Table S2. Teacher (n = 7) ratings for peer review event (1) (DOCX 22 kb) [file 12889_2019_7493_MOESM2_ESM.docx]

| **Additional File 2.**  **Supplementary Table 2. Teacher (n=7) ratings for peer review event (1)** | | | | |
| --- | --- | --- | --- | --- |
|  | To a great extent | To some extent | To a little extent | To no extent |
| I have gained new knowledge related to the provision of PE, physical activity or sport for girls in my school | 86% | 14% | 0% | 0% |
| I have improved my skills to help me undertake my role more effectively | 71% | 29% | 0% | 0% |
| I have been inspired and motivated to improve the delivery of PE, physical activity or sport for girls in my school | 86% | 14% | 0% | 0% |
| Do you feel supported by the Girls Active delivery team | 100% | 0% | 0% | 0% |
| Do you feel clear on your next steps | 86% | 14% | 0% | 0% |
| Are you in a position to take action | 71% | 29% | 0% | 0% |
