# Supplementary material for: Process evaluation of the school-based Girls Active programme
Source: BMC Public Health. 2019 Aug 29;19:1187. doi: 10.1186/s12889-019-7493-7 (PMC6716893; doi:10.1186/s12889-019-7493-7)
Supplement: Supplementary file 3 — Table S3. Teacher (n = 7) ratings for peer review event (2) (DOCX 22 kb) [file 12889_2019_7493_MOESM3_ESM.docx]

| **Additional File 3.**  **Supplementary Table 3. Teacher (n=7) ratings for peer review event (2)** | | | | |  |
| --- | --- | --- | --- | --- | --- |
|  | Very good | Good | Average | Poor | Very Poor |
| Supporting information and details sent in advance | 57% | 43% | 0% | 0% | 0% |
| Venue | 43% | 57% | 0% | 0% | 0% |
| The deliverers | 86% | 14% | 0% | 0% | 0% |
| Format of delivery | 86% | 14% | 0% | 0% | 0% |
| Pace of delivery | 86% | 14% | 0% | 0% | 0% |
| Meeting your needs | 86% | 14% | 0% | 0% | 0% |
| Overall, how would you rate your experience of the peer review workshop? | 86% | 14% | 0% | 0% | 0% |
